# Prevalence of Diabetes and Associated Obesity in Pennsylvania Adults, 1995–2010

**DOI:** 10.5888/pcd11.130330

**Published:** 2014-07-03

**Authors:** Oralia Garcia-Dominic, Eugene J. Lengerich, Fabian Camacho, Nancy R. Gallant, Linda A. Wray, Frank Ahern, Greg Bogdan, Gene Weinberg, Jan S. Ulbrecht

**Affiliations:** Author Affiliations: Eugene J. Lengerich, Fabian Camacho, The Pennsylvania State University and Penn State Hershey Cancer Institute, Hershey, Pennsylvania; Nancy R. Gallant, Linda A. Wray, Frank Ahern, The Pennsylvania State University, Hershey, Pennsylvania; Greg Bogdan, Gene Weinberg, The Pennsylvania Department of Health, Harrisburg, Pennsylvania; Jan S. Ulbrecht, The Pennsylvania State University and Penn State College of Medicine, Hershey, Pennsylvania.

## Abstract

**Introduction:**

This study examined trends in the prevalence and sociodemographic distributions of diabetes and the associations of diabetes with obesity over time in adult Pennsylvanians from 1995 through 2010.

**Methods:**

We used Behavioral Risk Factor Surveillance Survey data collected from 1995 through 2010. Diabetes prevalence was assessed by self-report of physician diagnosis. Obesity was assessed by body mass index computed from self-report of height and weight. State-level data for diabetes and associated obesity prevalence from 1995 through 2010 were collected for each year. Data on sociodemographic factors (age, sex, race, income, education) and 1 known disease risk factor (obesity) were also collected. Logistic regression modeling was used to examine associations between diabetes, sociodemographic factors, and obesity.

**Results:**

Diabetes prevalence in Pennsylvania, which increased from 5.6% in 1995 to 10.5% in 2010, followed national trends but exceeded the national prevalence each year by approximately 0.6 percentage points for 12 of the 16 years. The increase in prevalence was not equal across all socioeconomic groups. Obesity became a more dominant risk factor for diabetes during these 16 years.

**Conclusion:**

The burden of diabetes and obesity in Pennsylvania is substantial and increasing. Program managers and policy makers in Pennsylvania should consider these trends when allocating limited resources and designing programs for reducing diabetes-related illness. Other states may consider similar studies to monitor the prevalence of diabetes and determine whether disparities are changing and whether programs and resources should also shift.

## Introduction

The prevalence of type 2 diabetes is increasing in the United States; the increase primarily affects middle- and older-aged adults (1) but now also affects children (2,3). At the individual level, diet (4), physical inactivity (5), and obesity have been clearly linked to an increased risk of diabetes. However, at the population level, the increase in the prevalence of diabetes has not been linked to changes in the prevalence of risk factors over time.

The pathogenesis of insulin resistance and the broader metabolic syndrome are not completely understood; however, the Western diet, physical inactivity (6–8), and genetic factors appear to play central roles. The Western diet promotes excessive caloric intake, resulting in weight gain and often obesity, potentially leading to diabetes. Obesity aggravates insulin resistance. In the past 2 decades, obesity rates in the United States have reached epidemic proportions (9–11).

The Diabetes Prevention Program of the Pennsylvania Department of Health, in coordination with the Centers for Disease Control and Prevention (CDC), tracks diabetes and obesity prevalence in Pennsylvania through the annual Behavioral Risk Factor Surveillance System (BRFSS) (12). Although the BRFSS has demonstrated important differences in the prevalence of diabetes by sociodemographic groups (13,14), no studies have examined the relative importance of each risk factor in the prevalence of diabetes over time.

The objectives of this study were to examine trends in the prevalence of diabetes among adults in Pennsylvania and to determine whether the strength of the association between the prevalence of diabetes and the prevalence of risk factors changed over time. Because a major modifiable risk factor for diabetes is overweight/obesity (15,16), we also explored the change in the prevalence of obesity.

An understanding of the population-level relationships between diabetes and its risk factors will help policy makers and program managers develop population-level policies and public health programs that target risk factors for diabetes, an important strategy to prevent diabetes-related illness at the population level.

## Methods

### Study design, data source, and measures

The Pennsylvania BRFSS is a cross-sectional, annual, random-digit–dialed telephone survey conducted by the Pennsylvania state health departments and CDC among noninstitutionalized adults aged 18 years or older; data used for this study were collected from 1995 through 2010. All BRFSS survey data are self-reported. This study was approved by the Penn State Institutional Review Board.

The technical aspects of the BRFSS are described elsewhere (12). The sample size of the Pennsylvania BRFSS and the number of questions asked has increased over the years (12). Starting in 2001, the BRFSS allowed respondents to choose more than 1 race, which required a revision to the calculation of race variables (17). To ensure continuity across data years, we used 2 race variables: original reported race for 1995–2000 and preferred race for 2001–2010.

We collected and assessed data on diagnosis of diabetes, age, sex, race, height, weight, education, and annual income. A diagnosis of diabetes was determined by a response of yes to the question “Have you ever been told by a physician that you have diabetes?” Respondents reporting gestational diabetes were counted as not having diabetes, and we excluded their data from analysis. Age was coded as 18 to 44 (young [reference]), 45 to 64 (middle-aged), and 65 or above (older). Sex was coded as female (reference) and male. Race was coded as white (reference) and nonwhite. For height and weight, participants were asked “About how much do you weigh without shoes?” and “About how tall are you without shoes?” Body mass index (BMI) was calculated by using self-reported weight and height. BMI (kg/m^2^) was coded as normal weight (BMI 18.5–24.9 [reference]), overweight (BMI 25.0–29.9), and obesity (BMI ≥30.0). Education was coded as some college or more (reference), high school graduate, and less than a high school degree. Annual income was coded as more than $50,000 (high [reference]), $25,000 to $49,999 (middle), and less than $25,000 (low).

From 1995 through 2010, 121,867 adults participated in the Pennsylvania BRFSS; we excluded 2,241 adults because some of their data were missing; they reported gestational diabetes, prediabetes, or borderline diabetes; or they did not know or were not sure about their diabetes status. Our final sample consisted of 119,626 adults. Although people were invited to participate each year independently of previous years, there was a remote possibility that BRFSS surveyed the same person in more than 1 year (17).

### Periods, stratification, and handling of missing data

We examined trends in diabetes prevalence and BMI from 1995 through 2010 by year and by 3 periods: 1995–2000, 2001–2005, and 2006–2010. We compared the prevalence of diabetes and obesity in Pennsylvania with the national prevalence for each year (on the basis of national BRFSS data); we also calculated the approximate number of adults in Pennsylvania who had diabetes according to the prevalence of diabetes in Pennsylvania in 1995 and 2010 (18,19). 

Except for data for 2010, data for all years in each period were assessed as having the same sample design. Stratum (_STSTR) and primary sampling unit (_PSU) variables for each year were labeled differently to ensure nesting within year. Additionally, the weighting variable (_FINALWT) was divided by the number of years in the period. Unlike previous years, the 2010 BRFSS survey design did not include clustering. To incorporate 2010 data into the analysis, we assigned a unique clustering identification to each record before adding it into the data for 2006 through 2010.

Missing data in the covariates were a concern: data were missing from 20% of the unweighted records. Annual income accounted for 14% of the records with missing data; BMI accounted for 6%. For the multivariate analysis, we used the imputation and variance estimation software IVEware (Survey Methodology Program, Survey Research Center, Institute for Social Research, University of Michigan, Ann Arbor, Michigan) (20), which implements a sequential regression imputation method (21). The method incorporates complex survey designs and imputes categorical data by using polytomous regressions. Predictors in the imputation modeling included all the covariates used in the final multivariate analysis (age, sex, race, BMI, education, income, and period), the outcome variable (diabetes), the sampling design variables, and all possible pairwise interactions between covariates (ie, sex by age, sex by race, etc). Five separately imputed data sets were created and analyzed separately, and results were combined.

### Statistical analysis

Statistical modeling was performed by using SUDAAN 11.0.0 (RTI International, Research Triangle Park, North Carolina), which incorporates the sample design variables specific to the BRFSS survey. For the multivariate analysis, we fit a logistic regression model using proc RLOGIST to examine the predictive factors of diabetes and the interactions of these factors with each period. Independent variables included sex, age, race, BMI (treated continuously), education, income, and period. Interactions for each covariate with each period were examined manually by including them separately and sequentially in the model and testing for significance. Because SUDAAN allows for back transformation to the probability scale in the form of model-adjusted risk differences (RDs), which compare differences in the average marginal probabilities (22), and model-adjusted risk ratios (RRs) (23), which compare ratios of average marginal probabilities instead of differences, we included covariates with significant heterogeneity among the RDs across periods in the logistic model, even if interaction on the multiplicative scale was not significant. Because the BRFSS changed race variables in 2001, we compared only 2001–2005 with 2006–2010 for race.

## Results

The greatest percentage of respondents in the Pennsylvania BRFSS were aged 18 to 44 years old (50.1% in 1995–2000, 47.6% in 2001–2005, and 45.5% in 2006–2010), women (~52% in each period), and white (89.4% in 1995–2000, 87.2% in 2001–2005, and 86.4% in 2006–2010) (Table 1). Average BMI ranged from 29.7 in 1995–2000 to 31.8 in 2006–2010. Most respondents in each period had some college or more; only 12.0% in 1995–2000 and 7.8% in 2006–2010 had less than a high school degree. In 1995–2000, most respondents reported a middle annual income; in the later 2 periods, most reported a high annual income (Table 1). Diabetes prevalence was highest in all 3 periods among those who were aged 65 or older, nonwhite, and obese and among those did not have a high school degree. Diabetes prevalence was higher among women than among men only in 1995–2000; it was higher among men thereafter. Another shift in diabetes prevalence took place for annual income: in the first 2 periods, those with a low income reported the highest prevalence of diabetes among the 3 income categories. In 2006–2010, those with a middle income reported the highest prevalence of diabetes (Table 1).

**Table 1:** Characteristics of Pennsylvania BRFSS Study Respondents During 3 Periods, 1995–2000, 2001–2005, and 2006–2010^a^

Characteristic	1995–2000		2001–2005		2006–2010	
	All Study Respondents (N = 21,321)	Respondents Reporting Diabetes (N = 1,242)	All Study Respondents (N = 39,676)	Respondents Reporting Diabetes (N = 3,579)	All Study Respondents (N = 58,629)	Respondents Reporting Diabetes (N = 7,217)
**Demographic Factors**						
**Diabetes prevalence, %**	6.0	100	7.8	100	9.3	100
**Age, y**						
18–44, %	50.1	1.7	47.6	2.3	45.5	2.7
45–64, %	29.2	7.6	32.3	9.8	34.4	11.4
≥65, %	20.7	13.9	20.1	18.0	20.1	20.5
All, mean (SD)	61.3 (0.5)	—	61.2 (0.4)	—	61.5 (0.4)	—
**Sex**						
Male, %	47.3	5.7	47.9	7.9	48.3	10.0
Female, %	52.7	6.2	52.1	7.8	51.7	8.6
**Race**						
Nonwhite, %	10.6	7.4	12.8	9.4	13.6	13.0
White, %	89.4	5.8	87.2	7.6	86.4	8.7
**Risk Factors**						
**Body mass index, kg/m^2^**						
Normal (18.5–24.9), %	43.8	2.9	38.9	3.5	36.2	3.5
Overweight (25.0–29.9), %	37.2	6.1	37.4	7.3	36.6	8.1
Obese (≥30.0), %	18.9	12.8	23.7	16.2	27.2	18.5
All, mean (SD)	29.7 (0.2)	—	30.9 (0.2)	—	31.8 (0.2)	—
**Socioeconomic Status Factors**						
**Education**						
<High school degree, %	12.0	12.6	9.7	14.0	7.8	15.0
High school graduate, %	41.3	6.2	39.3	8.9	36.7	11.3
≥Some college, %	46.7	4.0	51.0	5.8	55.5	7.1
**Income, $**						
Low (<25,000), %	34.2	9.5	28.7	12.6	28.2	10.0
Middle (25,000–50,000), %	38.4	4.8	33.2	7.1	23.8	16.0
High (>50,000), %	27.4	3.1	38.1	4.4	47.9	5.5

Abbreviations: BRFSS, Behavior Risk Factor Surveillance System; SD, standard deviation; —, does not apply.

a Proportions are survey weighted.

**Figure 1:**
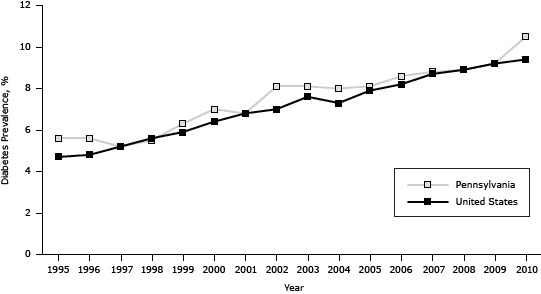
Prevalence of diabetes in the United States and among Pennsylvania adults, 1995–2010. Source: Behavioral Risk Factor Surveillance System. **Table:** 

The estimated prevalence of diabetes among Pennsylvania adults increased significantly from 5.6% in 1995 (approximately 12.1 million adults) to 10.5% in 2010 (approximately 12.7 million adults), generally paralleling the national trend, which increased significantly from 4.7% in 1995 to 9.4% in 2010 (*P* < .001). However, the prevalence in Pennsylvania was on average 0.6 percentage points higher (*P* < .001) than the prevalence in the United States (Figure 1). 

Year	Pennsylvania, %	United States, %
1995	5.6	4.7
1996	5.6	4.8
1997	5.2	5.2
1998	5.5	5.6
1999	6.3	5.9
2000	7.0	6.4
2001	6.8	6.8
2002	8.1	7.0
2003	8.1	7.6
2004	8.0	7.3
2005	8.1	7.9
2006	8.6	8.2
2007	8.8	8.7
2008	8.9	8.9
2009	9.2	9.2
2010	10.5	9.4

**Figure 2:**
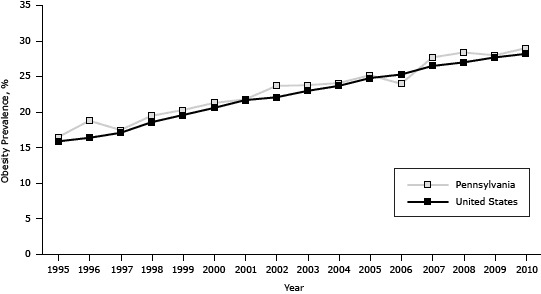
Prevalence of obesity among adults in Pennsylvania and the United States, 1995–2010. Source: Behavioral Risk Factor Surveillance System. **Table:** 

The prevalence of obesity in Pennsylvania increased from 16.5% in 1995 to 29.0% in 2010, generally paralleling the national trend (which increased from 15.9% in 1995 to 28.2% in 2010 [P < .001]) but was on average 0.8 percentage points higher (*P* < .001) (Figure 2). The association between diabetes and obesity was significant (odds ratio [OR], 1.1, *C* value = 0.7, *P* < .001) for each year from 1995 through 2010; the Wald test for interaction of BMI and year was *P* = .05.

Year	Pennsylvania, %	United States, %
1995	16.5	15.9
1996	18.8	16.4
1997	17.5	17.1
1998	19.5	18.6
1999	20.3	19.6
2000	21.3	20.6
2001	21.8	21.7
2002	23.7	22.1
2003	23.8	23.0
2004	24.1	23.7
2005	25.2	24.8
2006	24.0	25.3
2007	27.7	26.5
2008	28.4	27.0
2009	28.0	27.7
2010	29.0	28.2

The logistic regression showed significant associations between diabetes and age, sex, race, BMI, education, and income (Table 2). Diabetes prevalence was significantly higher (*P* < .001) for respondents aged 45 to 64 (OR, 4.4; RR, 3.8; RD, 6.6 percentage points) and 65 or older (OR, 9.3; RR, 6.8; RD, 13.9 percentage points) than for respondents aged 18 to 44. The likelihood of reporting diabetes increased by 1.1 (*P* < .001) for each unit increase in BMI. When we compared a BMI of 27 (sample average) with a BMI of 26, RR was 1.1, and RD was 0.6 percentage points (*P* < .001). Respondents who did not graduate from high school were more likely (OR, 1.2; RR, 1.2; RD, 1.3 percentage points, *P* < .001) to report diabetes than were adults with at least some college, but high school graduates were not more likely than adults with at least some college to report diabetes. In each period, respondents with low or middle income were more likely to report diabetes than respondents with high income.

**Table 2:** Selected Factors Associated With Diabetes in BRFSS, Pennsylvania Adults With Diabetes, 1995–2010 Combined Data Set^a^

Variable	OR (95% CI)	*P* Value for OR	Model-Adjusted Marginal Risk Ratio (95% CI)	Model-Adjusted Marginal Risk, Percentage-Point Difference (95% CI)	*P* Value for Risk Difference
**Demographic Factors**					
**Age, y**					
18–44	1 [Reference]						
45–64	4.4 (4.0–4.9)	<.001	3.8 (3.4–4.1)	6.6 (6.2–7.0)	<.001
≥65	9.3 (8.4–10.3)	<.001	6.8 (6.2–7.4)	13.9 (13.2–14.6)	<.001
**Sex**					
Female	1 [Reference]				
1995–2000 Male	1.1 (1.0–1.3)	.15	1.1 (1.0–1.2)	0.6 (−0.1 to 1.3)	.15
2001–2005 Male	1.3 (1.1–1.4)	<.001	1.2 (1.1–1.3)	1.5 (0.8–2.2)	<.001
2006–2010 Male	1.5 (1.3–1.6)	<.001	1.3 (1.3–1.4)	2.7 (2.1–3.3)	<.001
Wald test of interaction, *P* value^b^	.005		—	<.001	
**Race**					
White	1 [Reference]				
1995–2000 Nonwhite	1.4 (1.1–1.7)	.005	1.3 (1.1–1.6)	1.8 (0.4–3.2)	.009
2001–2005 Nonwhite	1.4 (1.2–1.7)	<.001	1.3 (1.2–1.5)	2.5 (1.2–3.8)	<.001
2006–2010 Nonwhite	1.8 (1.6–2.2)	<.001	1.6 (1.4–1.8)	4.9 (3.3–6.5)	<.001
Wald test of interaction, *P* value^c^	.04		—	.02	
**Risk Factors**					
**Body mass index (BMI)**	1.1 (1.1–1.1)	<.001	1.1 (1.1–1.1)^d^	0.6 (0.6–0.6)^d^	<.001
**Socioeconomic Status Factors**					
**Education**					
≥Some college	1 [Reference]				
High school graduate	1.0 (1.0–1.1)	.36	1.0 (1.0–1.1)	0.2 (−0.3 to 0.7)	.36
<High school degree	1.2 (1.1–1.4)	<.001	1.2 (1.1–1.3)	1.3 (0.6–2.0)	<.001
**Income^e^**					
High	1 [Reference]				
1995–2000 Middle	1.3 (1.1–1.7)	.02	1.3 (1.0–1.6)	1.3 (0.2–2.4)	.02
2001–2005 Middle	1.4 (1.2–1.6)	<.001	1.3 (1.1–1.5)	1.7 (0.9–2.5)	<.001
2006–2010 Middle	1.4 (1.2–1.6)	<.001	1.3 (1.2–1.5)	2.0 (1.2–2.8)	<.001
Wald test of interaction, *P* value	.98^f^		—	—	.43^g^
1995–2000 Low	1.8 (1.5–2.3)	<.001	1.7 (1.4–2.1)	3.1 (2.0–4.2)	<.001
2001–2005 Low	1.9 (1.7–2.2)	<.001	1.7 (1.5–2.0)	4.1 (3.2–5.0)	<.001
2006–2010 Low	2.0 (1.7–2.3)	<.001	1.7 (1.7–2.0)	4.8 (3.9–5.7)	<.001
Wald test of interaction, *P* value	—		—	.01^g^	

Abbreviations: BRFSS, Behavior Risk Factor Surveillance System; OR, odds ratio; CI, confidence interval; —, does not apply.

a Logistic regression average *C* = 0.8; average Cox and Snell *R* ^2^ = 1.0. All variables in Table 2 were included in the logistic regression/linear probability models: (diabetes) = *f*(sex, age, race, obesity, education, income, year, sex × year, race × year, income × year). Parameter estimates were obtained by conducting multiple imputations, separately analyzing 5 imputed data sets, and combining results.

b *P* value is from a Wald test of interaction, or homogeneity, of sex odds ratios (risk differences) across 3 periods.

c *P* value is from a Wald test of homogeneity of race odds ratios (risk differences) across 2 periods. Only 2001–2005 and 2006–2010 were compared because of difference in definitions for race before 2001.

d Risk ratios and differences compare a BMI of 27 to a BMI of 26 only.

e Annual income categorized as high (>$50,000); middle ($25,000–$50,000); and low (<$25,000).

f *P* value is from a Wald test of interaction for income odds ratios across 3 periods.

g *P* value is from a Wald test for linear trend of marginal risk differences. Null hypothesis, H_o_: β = 0, assumes no linear trend.

Tests of interaction across the 3 periods showed varying associations of diabetes with sex and race (Table 2). Wald tests for interaction between sex and period were significant when we compared ORs and model-adjusted marginal RDs. Both measures suggested a monotonic increase from 1995–2000 to 2006–2010. Men had an increasingly greater likelihood of reporting diabetes than did women: ORs were 1.1 for 1995–2000, 1.3 for 2001–2005, and 1.5 for 2006–2010 (*P* = .005); RDs were 0.6 percentage points for 1995–2000, 1.5 percentage points for 2001–2005, and 2.7 percentage points for 2006–2010 (*P* < .001). Likewise, nonwhites had an increasingly greater likelihood of reporting diabetes than did whites: ORs were 1.4 for 2001–2005 and 1.8 for 2006–2010 (*P* = .04); RDs were 2.5 percentage points for 2001–2005 and 4.9 percentage points for 2006–2010 (*P* = .02).

Although we found no interaction between income and year on the multiplicative scale (*P* value for Wald test for homogeneity of ORs = .98), we found a significant interaction on the additive scale. A Wald test of the linear trend of the marginal RDs suggested that the low-income group had increasingly greater rates of diabetes than the high-income group: RDs increased from 3.1 percentage points in 1995–2000 to 4.1 percentage points in 2001–2005 and to 4.8 percentage points in 2006–2010 (*P* = .01). 

## Discussion

In Pennsylvania, the prevalence of diabetes increased from 5.6% in 1995 to 10.5% in 2010, paralleling the increase in the prevalence in the United States. However, the prevalence in Pennsylvania was higher than the prevalence in the United States for 12 of the 16 study years. Similarly, the increase in the prevalence of obesity in Pennsylvania paralleled the increase in the United States; for 15 of the 16 years the prevalence of obesity in Pennsylvania was greater than the prevalence in the United States. Public health and medical programs in Pennsylvania should take additional measures to reduce the rates of diabetes and obesity to rates that are below national rates.

Men were more likely than women to report diabetes in the most recent 2 periods, which is consistent with findings from other health surveys (17,24). Our data showed significant differences by sex in 3 measures (ORs, RRs, and RDs) for these 2 periods. These findings highlight the need to address diabetes and its risk factors among men, a group traditionally difficult to reach through public health strategies; innovative strategies may be warranted. Community-based interventions that focus on education delivered by trained lay health educators to improve physical activity levels is a potential strategy for men who have diabetes (25), and it is a strategy recognized by CDC (26). Similarly, the relatively greater increase in the prevalence of diabetes over time among nonwhites than among whites suggests the need to increase attention among public health policy makers and program managers toward nonwhite populations.

The prevalence of obesity was higher among Pennsylvania adults with low levels of education and income than among those with middle or high levels of education and income; similar findings have been reported elsewhere (27,28). We did not find that the prevalence of obesity changed by education level over time. However, the RDs associated with low income increased over time, suggesting that the prevalence of obesity increased faster among low-income respondents than among those with middle or high incomes.

In all 3 periods, the prevalence of diabetes was highest among those who were obese. Given the underlying alteration in glucose metabolism among obese people, this finding would be expected. Indeed, obesity was a dominant risk factor for diabetes over time in our study. However, unlike the changes by sex and race, the strength of association between population-level BMI and diabetes did not change. This lack of change in association would be expected if the major mechanism for pathogenesis had not changed over time.

Because diabetes is a lifestyle disease, lifestyle interventions should reduce risk. Several landmark studies have demonstrated lifestyle-related risk reduction ranging from 32% to 58% (5,29,30). However, in 2013, the Look AHEAD Research Group found no reduction in cardiac risk over time for people with diabetes in spite of behavioral modifications (31), highlighting the difficulties in implementing these interventions.

Many facets of government policy and action affect the environment in which people live and the behavior of individuals and therefore their risk for diabetes: public transportation and urban planning and zoning policies (through effects on physical activity); agricultural policy (through effects on, for example, food availability, price, or labeling); school food programs and in-school leisure time and physical education classes; and regulation and financing of health care and public health programs. A full discussion of these issues is beyond the scope of this article. However, congruent with the objective of this study is the identification of efforts nationally and in Pennsylvania to reduce diabetes risk through targeted programs. Policy issues related to lifestyle intervention can be grouped into 4 categories: 1) identification of people for lifestyle interventions (ie, all people vs people at high-risk), 2) methods to deliver lifestyle interventions within the health care system, 3) personal economics, and 4) ethics of selection criteria (28).

Several national campaigns directed at diabetes prevention and improvements in outcomes have been conducted (26). Common techniques used in these campaigns include print media, tool kits, news media, advertisements, curricula, direct mail, and telemarketing (26). Community-based diabetes programs that use lifestyle coaches or navigators or that have obesity-related components offering healthful food options, opportunities for parental involvement, and physical activity are also recognized by CDC (26). 

Diabetes prevalence and costs are increasing in Pennsylvania (32), and the epidemic of obesity coupled with an aging population will increase the burden. The *Pennsylvania Diabetes Action Plan 2010 *— based on diabetes-related surveillance data provided in the *Pennsylvania Diabetes 2007 Burden Report* — recommends strategies to lessen the burden (13,14). These strategies include surveillance, changes to health policy, and program evaluation. Our study may help to inform these strategies.

This is the first study to examine trends in the prevalence of diabetes, the association of diabetes with selected risk factors, and variations in the strength of associations from 1995 through 2010. Although the BRFSS is used in all 50 states plus the District of Columbia and US territories (it is the world’s largest ongoing health survey, with more than 1 million participants to date), it is not without limitations. First, BRFSS data on diabetes are based on self-report, which may cause bias (17); blood glucose levels are not measured. Second, BRFSS participants are noninstitutionalized adults aged 18 or older; therefore, the data set excludes children, adolescents, and people who are institutionalized, such as those living in nursing homes. Third, the BRFSS is a telephone survey; people who do not have telephones cannot participate. Fourth, the BRFSS is a repeated cross-sectional study and does not follow the same participants over time; therefore, it is not known whether any participants improve their diabetes status through lifestyle changes such as diet, exercise, or other health behaviors. Fifth, BRFSS data do not allow assessment of environmental factors that may influence the prevalence of diabetes (eg, parks and recreation centers, fitness and wellness facilities, workplace wellness programs, types of food establishments). Despite these shortcomings, the BRFSS is one of the best tools for monitoring health indicators. 

The burden of diabetes and obesity in Pennsylvania is substantial and increasing. Our study demonstrates that the increase in diabetes prevalence is not equal across all sociodemographic groups. Program managers and policy makers in Pennsylvania should consider these trends when allocating their limited resources and designing programs for reducing diabetes-related illness. Other states may consider similar studies to determine whether disparities are changing and whether programs and resources should also shift.
